# A Literature Review of Minimally Invasive Endodontic Access Cavities - Past, Present and Future

**DOI:** 10.14744/eej.2022.62681

**Published:** 2022-03-07

**Authors:** Maggie Yuk Ching CHAN, Venetia CHEUNG, Angeline Hui Cheng LEE, Chengfei ZHANG

**Affiliations:** From the Department of Restorative Dental Sciences (C.Z. 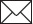 zhangcf@hku.hk, M.Y.C.C., V.C., A.H.C.L.), The University of Hong Kong, Faculty of Dentistry, Hong Kong, China

**Keywords:** Access cavity, fracture resistance, minimally invasive, root canal treatment, tooth preservation

## Abstract

Minimally invasive endodontic access cavities have gained popularity in academic discussions for their clinical applications in recent years. Although some studies showed an improved fracture resistance of endodontically-treated teeth accessed with a minimally invasive access cavity design, the resulting effectiveness and efficiency of subsequent root canal treatment procedures may be impaired. Aspects that may be impaired are canal detection and negotiation, chemomechanical debridement of the root canal system, quality of the obturation. These are potentially complicated by the increased incidence of procedural mishaps and compromised aesthetic outcomes. In addition, the inherent flaws presented in the methodology of some in vitro studies and the lack of a universal classification system are also of concern. This literature review aims to present a comprehensive overview of the development of the minimally invasive endodontic access cavity and summarise the currently available from a clinical context.

**HIGHLIGHTS**

• Different designs of minimally invasive access cavities have been proposed to improve the fracture resistance of the endodontically treated teeth by preserving the tooth substance of the pericervical dentine and the roof of the pulp chamber.
• Currently, the available evidence, mainly laboratory studies, has shown some improvement in fracture resistance in posterior teeth with MIECs. However, with the potential risks of procedural impairment, the use of MIECs is yet to be recommended universally. Proper training and armamentarium such as OM and heat-treated NiTi instruments may be prerequisites of clinical application.
• A universal classification system and consistent methodologies in future studies are required to validate the use of MIECs.

## INTRODUCTION

The contemporary practice of endodontics and restorative dentistry has shifted to preserving tooth structure. Minimally invasive endodontics refers to a concept that advocates the preservation of as much natural tooth structure as possible by downsizing the preparation of the access cavity, the taper of prepared canals, and the prepared apical size (1). This change was made possible by the availability of advanced endodontic armamentarium, such as cone beam computed tomography (CBCT), operating microscope (OM), and ultrasonic instruments (2).

Minimally invasive endodontic access cavities (MIECs) have been described as openings to gain access to the root canal system, which aim to preserve sound tooth structure. Common approaches to the preparation of MIEC are known as: (i) contracted access, (ii) "ninja" access, and (iii) "truss" access (3, 4). Advocates of these approaches believe that MIEC would help maintain the long-term survival of the endodontically-treated teeth (ETT) by avoiding unnecessary dentine removal, thus increasing the resistance of ETT against tooth fracture (4, 5). While the claim of preventing tooth fracture has yet to be clinically validated, there have been concerns regarding the potential drawbacks of MIEC approaches. For instance, a constricted access cavity design poses challenges in the subsequent procedural steps, including an impaired vision of the pulp chamber and canal, reduced effectiveness and efficiency in canal instrumentation and disinfection, and the loss of orientation (6-8). More research is warranted as it remains controversial whether the benefits postulated outweigh the potential drawbacks.

This literature review aims to summarise the advantages and disadvantages of MIEC based on the currently available evidence.

### Methodology

A structured literature search was performed involving electronic searches on PubMed (covering the time frame from 1966 to 2020), EMBASE (1947 to 2020), and Web of Science (1956 to 2020). Search terms applied were (Endodontic OR "root canal") AND ("access cavity" OR "access cavities") AND ("minimally invasive"). The abstracts of the articles obtained were reviewed independently by 2 reviewers. Articles included were limited to those written in English. After the removal of duplicates, 20 articles were retrieved. Additional articles were identified by reviewing the reference section of studies identified for the full-text review. A total of 44 studies were included for the review session on MIEC.

### Traditional concepts

**Figure 1.**
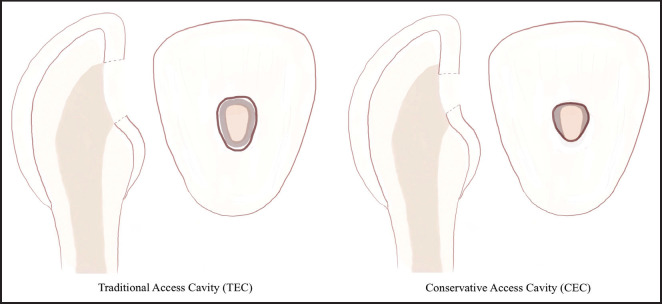
Schematic illustrations of different access cavity designs in a maxillary incisor

**Figure 2.**
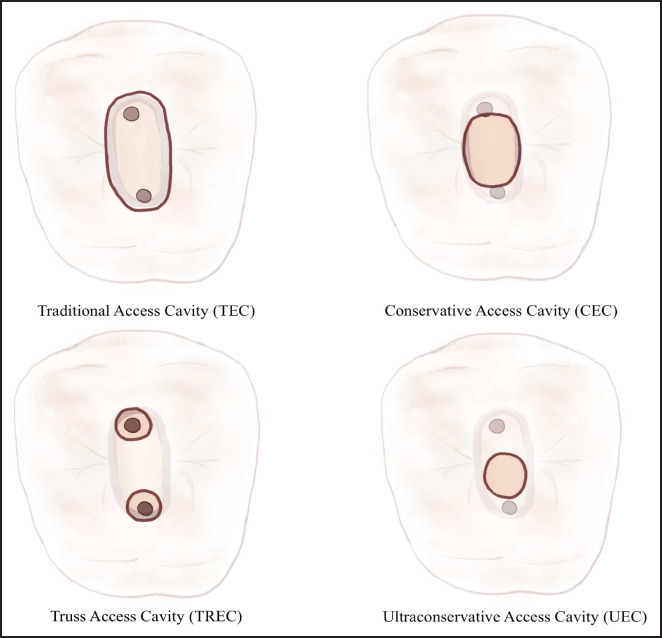
Schematic illustrations of different access cavity designs in a maxillary first premolar

**Figure 3.**
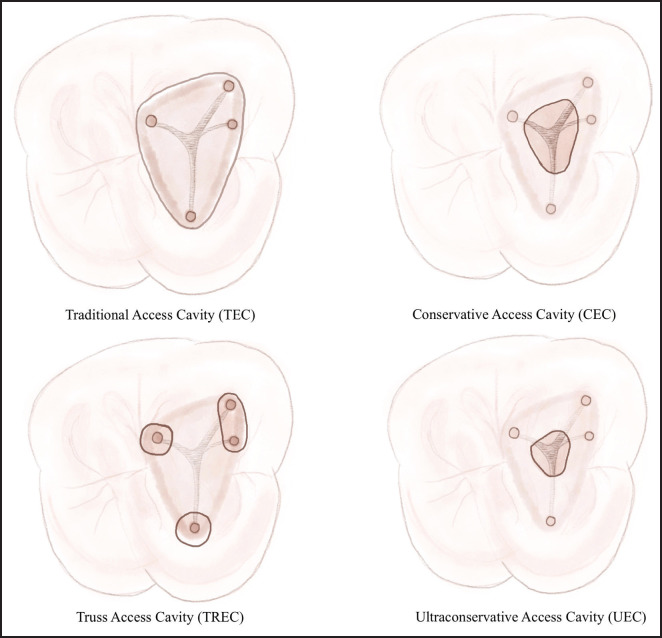
Schematic illustrations of different access cavity deisngs in a maxillary first molar

**Figure 4.**
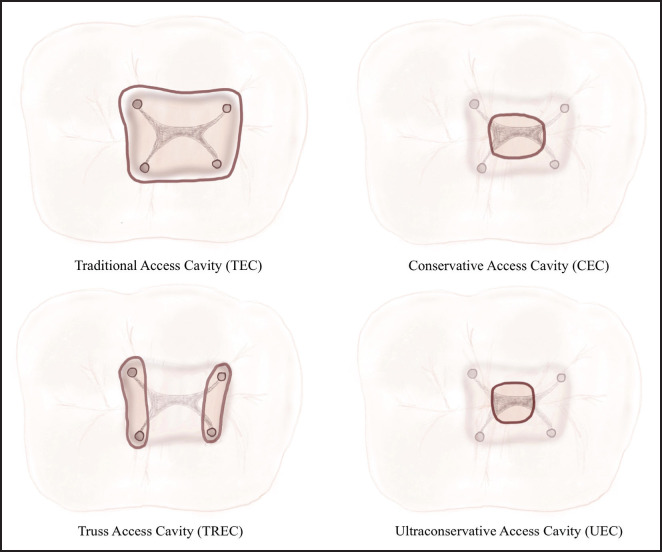
Schematic illustrations of different access cavity designs in a mandibular first molar

The importance of access cavity has been well validated as one of the key steps towards successful non-surgical root canal treatment. An adequately prepared access cavity facilitates the performance of subsequent clinical steps, including the detection of the canal orifice, chemomechanical debridement, obturation of the root canal, and reducing the chance of iatrogenic damage (9). One of the requirements of a traditional endodontic access cavity (TEC) is to allow for a straight-line introduction of the endodontic instruments into the canals without interference (Figs. 1-4) (10). To achieve this goal, an adequately extended access cavity by selective removal of the tooth structure is necessary. For example, the TEC approach typically requires removing the entire roof of the pulp chamber (9).

Non-surgical root canal treatment is a predictable treatment modality for preserving the natural dentition. Studies reported that the ETT fared similar survival rates to the implant-supported crowns, while the longevity of ETT appeared superior to the fixed dental prostheses (11). However, the survival rate of ETT can be jeopardised by their increased susceptibility to fracture due to the loss of tooth structure (12). This failure often results in the extraction of the ETT, a cause of frustration and disappointment to both the patients and clinicians (13).

The access cavity preparation has been shown to contribute to the loss of tooth structure during non-surgical root canal treatment (14). An excessive loss of sound tooth structure may cause a significant decrease in the fracture resistance and increased cuspal flexure of ETT under functional loading (15). Undoubtedly, the compromised structural integrity of ETT is known to be one of the key factors resulting in tooth fractures (16). Therefore, advocates to maximise tooth structure preservation during treatment have been the driving force behind the change in modern endodontics. Adjustments to the form and size of access cavity, canal taper, and apical preparation size have correspondingly been proposed (17, 18).

### Current developments

The concept of MIEC underlies the development of the conservative endodontic access cavity (CEC). All defective restorations and caries are removed before the preparation of CEC, as in the TEC (5, 19). However, in CEC, the remaining sound tooth structures are preserved more than the TEC by preparing the access cavity from the central fossa and extending only as far as needed to locate the canal orifices instead of gaining complete straight-line access to them (5). In addition, the axial walls of CEC are often slightly convergent and occlusally bevelled to allow for better visualisation of the pulp chamber and the canal orifices when viewed from different angles (20).

In addition to a more constrained occlusal outline in the CEC than TEC, the CEC also preserves part of the pulp chamber roof and pericervical dentine (PCD), the tooth substance 4 mm above and 4 mm apical to the alveolar bone crest (5). Preservation of the PCD structure appeared to be crucial for distributing the occlusal load from the occlusal table to the root (21). In some finite element analysis (FEA) studies, the maximum strain was shown at the cervical third of the teeth (21-23). Furthermore, the use of Gates Glidden (GG) burs for coronal enlargement and burs for the removal of pulp chamber roof was claimed to be detrimental to the structural strength of PCD and soffit (5). In fact, a few studies have demonstrated slight superiority of CEC over TEC, claiming that it offered the benefit of increased fracture resistance to the ETT by preserving the PCD and soffit (4, 20, 24). However, the concept of preserving the PCD in MIEC may only seem relevant as far as the "anatomical" crown of teeth with normal alveolar bone level is concerned. The teeth with loss of periodontal attachment and reduced alveolar bone height, as commonly seen in those with periodontal diseases, naturally result in an increase in the "clinical" crown height along with the apically positioned PCD (5).

Taking the conservative approach to a greater extent, an ultraconservative endodontic access cavity (UEC), also known as "ninja" access, was further proposed (Figs. 2-4). The UEC constitutes a design with an extreme preservation of the pulp chamber roof and forms severely convergent walls (Figs. 2-4) (25). The UEC creates a highly constricted access cavity merely aiming to locate the root canal orifices, thereby preserving a large portion of the pulp horns and occlusal enamel intact (4). Another variation of the constricted cavity design has also emerged, which is commonly known as the "truss" endodontic access cavity (TREC) (26). The TREC design involves the preservation of a dentine bridge and overlying enamel between separate cavities that are prepared to aim directly at the canal orifices in multi-rooted teeth, hence is also known as "orifice-directed dentine conservation access" (Figs. 2-4) (4, 26, 27).

Access cavity designs that employ minimally invasive principles are gaining popularity among clinicians (28). A survey recently conducted among members of the American Association of Endodontists revealed that 43% of the respondents adopted a "conservative" access cavity approach, while 57% used the "traditional" approach (28). Only 0.7% of the respondents reported using the "ultraconservative" access preparations (28). However, the option of a "conservative" access cavity was not well defined in the survey, thus leaving uncertainty as to whether the 43% of respondents that adopted the approach had the same understanding in mind and prepared the access cavity in the same specific way.

### Classification

To date, there is a lack of a universal classification system for the different designs of MIEC. For example, the definitions of CEC and UEC lack clear mutual exclusiveness thus may overlap in meaning. Current terms used in the literature (and their abbreviations) such as "conservative", "contracted", and "ultraconservative" do not offer precise quantitative categorisation. Their usage is often empirical and not surprisingly may have been used interchangeably by some. It may be confusing to understand the exact extension and features of the access cavity designs adopted in the studies (3, 25). Apart from this, variations in the tooth morphology and operator experience and skills are also factors that might have hindered the development of a standard nomenclature that can encompass all MIEC designs. Despite these challenges, attempts have been ongoing to develop new classification to bridge the communication gap amongst the researchers and clinicians by using a set of consistent terminologies.

Several authors have proposed the classifications based on the anatomical landmarks projecting to the occlusal surface, design principles, and the percentage of the volume of tooth structure removal. Based on the micro-CT evaluation, Eaton et al. (29) proposed 3 types of access cavity design based on the landmarks of the root canal system in the mandibular molars, namely "minimally invasive", "straight-line furcation", and "straight-line radicular". Silva et al. (3) identified some abbreviations and terms used in selected literature to classify the different types of access cavity geometries. An attempt was made to consolidate 20 out of 22 of them into 6 main categories, which are (i) traditional access cavity, (ii) conservative access cavity, (iii) ultraconservative access cavity, (iv) truss access cavity, (v) caries-driven access cavity, and (vi) restorative-driven access cavity (3). Isufi et al. (25) also introduced a quantitative approach to measure the tooth substance loss using CBCT and micro-CT imaging based on the percentage of the volume of dentine and enamel removal (DER). The authors reported that the DER of TEC, CEC and UEC in the molars and premolars was >15%, ≤15%, and ≤6%, respectively.

The proposed classification and quantitative measurement method aim to facilitate future research studies on the different types of access cavities using standardised measures (25). However, some authors might argue that such standardisation may be more of a matter of academic interest to facilitate communication (3). In the context of clinical application, CEC is said to be a vision-based and stepped access with a strategic extension (5, 19). Therefore, it should embrace the individuality of teeth with variable anatomy and morphology instead of an absolute outline form (5, 19). Most available studies were conducted on intact teeth in laboratory settings (4, 6-8, 20, 24, 26, 27, 29-62). Thus, questions remain about the application of MIEC in carious, heavily restored, and crowned teeth (63). One of the main indications of root canal treatment is pulpal or periapical pathology resulting from deep caries (64). After removing caries and existing restorations, it is often possible to access the pulp chamber as an extension of the prepared cavity. However, the authors speculate that preservation of the soffit and PCD might not always be possible when the carious lesion or existing cavity have already jeopardised the structural integrity of the teeth in these critical areas, hindering the application of CEC. As a result, the amount of additional tooth substance removal might not differ much between TEC and CEC. As currently available evidence is mainly based on intact teeth (4, 6-8, 20, 24, 26, 27, 29-62), more studies will be needed further to consolidate the idea of MIEC in clinically relevant applications.

### Armamentarium

The availability of nickel-titanium (NiTi) engine-driven instruments, OM, ultrasonic instruments and CBCT has made the MIEC approach potentially feasible in clinical endodontics (47, 56, 58). These technological advancements have enhanced operators' vision and improved precision handling of instruments, making the need to achieve straight-line access by extending the access cavity to a certain outline form less important (34).

#### Access burs

In general, small-sized tip and long-shank burs could be used to enhance visibility and preservation of tooth structure (5, 19). In studies investigating the MIEC design, spherical diamond burs such as the spherical diamond tip FG 1012 (KG Sorensen, Barueri, SP, Brazil) and tapered round-end diamond bur such as the torpedo diamond bur FG 856 (Komet Italia Srl, Milan, Italy) were often used (4, 6, 8, 27, 31, 34, 35, 51, 57).

#### Canal preparation instruments

Traditional step-down or step-back instrumentation techniques commonly use GG burs and Peeso reamers for coronal flaring to establish straight-line access (65). Both GG burs and Peeso reamers are aggressive by indiscriminately enlarging the canals (66). Increased incidence of furcation strip perforation and canal transportation has been associated with the injudicious use of these instruments (67). The advancement in engine-driven NiTi instrument design and alloy treatment has given rise to NiTi files with enhanced super-elasticity, shape memory, and cyclic fatigue resistance compared to their traditional counterparts and stainless steel instruments. Furthermore, NiTi instruments also facilitate well-centred canal preparation (67), enabling better conformation to the original path of narrow and curved canals (68).

Some authors stressed the importance to use the NiTi files that have undergone thermal treatment in MIEC (30, 42, 43, 52, 53). Research has attributed the non-occurrence of instrument fractures in preparing MIEC to the use of heat-treated NiTi instruments (30, 42).

#### Operating microscope and ultrasonic instruments

The use of an OM is indispensable when performing MIEC, which is supported by the majority of studies utilising OM when comparing the effects between TEC and MIEC (6-8, 20, 31, 32, 35, 39, 40, 42, 47, 54-58). The excellent magnification and illumination of the OM offer the benefits of improved vision and ergonomics for the operator (69). The OM allows for direct visualisation of the entire pulp chamber and easier identification of the anatomical landmarks, such as the developmental grooves on the pulp chamber floor and the subtle colour difference reflected from the dystrophic calcific structures (70). It has been shown that there was a significantly higher chance of locating MB2 and additional canals in the maxillary molars when the operators worked with an OM compared to those without (71).

Ultrasonic instruments are also important adjuncts in the preparation of MIEC as they enable effective debridement, precise and selective removal of the obstructions such as pulp stone, and direct visual examination of the pulp chamber floor, significantly improving the ability of the operators to detect extra canals (72). Plotino et al. (73) stated that the treatment outcome and predictability of root canal treatment could be improved with the conjunctive use of OM and ultrasonic instrumentation. This is supported by Rover et al. (7) as no significant differences in root canal detection in the maxillary molars between the TEC and CEC groups was demonstrated when OM and ultrasonic troughing were conjunctively used. However, the use of OM alone offered no significant improvement in canal detection for the CEC group when compared to the TEC groups, reinforcing the importance of the conjunctive use of OM and ultrasonic troughing (7). Lara-Mendes et al. (74) also found that the same level of success in locating mid-mesial canal in the mandibular first molars for TEC groups could be achieved by the CEC group when OM and ultrasonic troughing were used in combination.

### Effects of MIEC on non-surgical root canal treatment

While the effect of MIEC on the fracture resistance of ETT remains debatable, a few possible drawbacks caused by an inadequately extended access opening were mentioned in the literature (3, 7, 20, 26, 30, 32, 40, 52, 55). The problems associated with a constricted access include:

(i) The ability of canal detection and negotiation (3, 7, 31, 56);
(ii) The quality of chemomechanical preparation, obturation and post-endodontic restoration (6-8, 20, 26, 32, 42, 43, 45, 47, 55);
(iii) Increased iatrogenic mishaps (6, 7, 20, 30, 39, 42, 43, 47, 52, 53, 55);
(iv) Negative effects on the aesthetic outcome (40, 55, 75); and,
(v) Prolonged treatment time (6, 8, 39, 40, 52).

#### Fracture resistance of the ETT

In the past, studies have mainly focused on the effect of MIEC on the fracture resistance of ETT, as this improved biomechanical property was considered the foremost important benefit offered by the constricted access cavity (4, 7, 20, 24, 27, 33, 34, 38, 42, 44, 55, 57). The first report by Krishan et al. (20) showed a higher fracture resistance of ETT prepared with CEC than those with TEC in the mandibular premolars and molars. However, interpretation of the results of this study should be taken with caution because specimens used were subjected to experimental loads without the presence of post-endodontic restorations. In a classical *in vitro* study, Reeh et al. (1989) (76) showed that the ETT restored with small direct composite restorations had the fracture resistance comparable to intact teeth.

In order to reproduce the actual clinical scenario, a few recent studies have tested their experimental specimens in the presence of post-endodontic restorations and continued to demonstrate the superiority of the fracture resistance of ETT prepared with CEC in both the premolars and molars when compared to TEC (4, 24). In the meantime, other studies showed no statistically significant differences in the fracture resistance between the CEC- and TEC-prepared teeth, including the maxillary premolars (38, 44), mandibular premolars (44), upper molars (7, 34, 42), and lower molars (27, 33, 55, 57).

In summary, there are no studies investigating the effects of CEC on the fracture resistance of ETT in the anterior teeth, while the fracture resistance of ETT in the posterior teeth accessed with CEC were found either comparable to or better than those accessed with TEC (20, 43). A recently published systematic review also concluded that there was no strong and high-quality evidence backing the shift of the current clinical practice to MIEC (77).

The mode of failure was also investigated in many studies. It is well accepted that a catastrophic failure of ETT often leads to extraction, while a more favourable and restorable fracture pattern yields a better chance for tooth survival (62). Cuspal chipping, which is deemed more favourable, was observed in the mandibular premolars accessed with CEC, while catastrophic cuspal fracture was more frequently occurred in TEC (20). Özyürek et al. (33) also noted that the mandibular first molars prepared with the CEC and restored with Class II composite restoration had significantly more restorable fractures than those prepared with the TEC, despite no significant differences found in the fracture strength between these two types of access cavity design. This signified the positive influence of CEC on the fracture mode of ETT. However, other studies reported comparable rates of restorable and unrestorable fractures between the TEC and CEC groups in all the premolars and molars (4, 6, 8).

There are relatively fewer studies that explored the effect of TREC on ETT when compared to CEC. All the studies reviewed only studied the mandibular molars. While some observed improved fracture strength of teeth prepared with TREC (35, 51), others found no significant differences between the TEC and TREC (27, 55). It is noteworthy that among these studies, only one had simulated the *in vivo* condition by subjecting the experimental specimens to thermocycling in order to mimic the thermal alterations in the oral cavity (51).

Some other researchers have studied the effect of UEC on the fracture resistance of ETT (4, 6, 8). For example, Plotino et al. (4) observed an increased fracture strength in the premolars and molars accessed with UEC compared to TEC, while no significant differences were found between the UEC and CEC. However, to our knowledge, this is the only study that offered a comparison between the TEC, CEC and UEC to date. Meanwhile, no studies have found any differences in the fracture strength between the UEC and TEC (6, 8).

The conflicting findings might be attributed to the differences in the methodological design, including the number of samples, tooth types studied, presence or absence and the type of restoration, and the design of fracture testing parameters (4, 6, 27, 35, 53, 55). In their recently published review article, Silva et al. have thoroughly described the risk factors that may cause variability and discrepancies in the research findings, as well as confounding the reliability of the studies. One of the risk factors includes tooth ageing, which was said to reduce the toughness and ductility of the sampled teeth (3). In addition, the difference in the crown and root morphology was identified as another risk factor that should not be ignored (3). The authors have made some suggestions to overcome these problems. Firstly, using the three-dimensional imaging tools such as micro-CT or CBCT was recommended to reduce the heterogeneity in sample selection (3), as anatomical matching based on the external and internal anatomy will be made possible with the use of these advanced imaging technologies. In addition, confounding factors, such as the differences in the pulp chamber volume, and the height and volume of the remaining tooth structure, can also be minimised. Secondly, detailed reporting of the parameters to improve the validity of studies was also proposed, including tooth age, extraction technique, storage condition, sample handling immediately after extraction, and sample preparation. Finally, the use of finite element analysis (FEA) was advocated. A number of studies that used the FEA demonstrated that teeth accessed with MIEC showed less stress concentration in the PCD area in the upper first molars (36, 37, 49); while an increased fracture strength was also found in the upper first molar (36, 49) and lower first molar (41, 59).

To reiterate, a universal classification of MIEC is of paramount importance, as it allows comparison of the findings by reducing heterogeneity between studies and facilitates communication amongst the researchers. Augusto et al. (6) pointed out that confusion in the classification of different types of cavity design could be an underlying cause of the conflicting results obtained in different studies. Saberi et al. (51) accounted for the differences between their findings with Moore et al.'s (42) due to methodological differences. However, it has been noted that the access cavity designs investigated in the two studies were different; that is, TREC in the former (51) and contracted endodontic access cavity that appeared analogous to UEC in the latter (42).

#### Canal detection and negotiation

The ability of canal detection in MIEC was shown to be highly dependent on OM and ultrasonic instruments (7). It was found that a similar rate of canal detection could be expected in the CEC when compared to the TEC, given that the OM was used in conjunction with the ultrasonic devices (3, 31, 56). It was speculated that the detection of extra canals is affected by diagnostic aids more than the cavity design (56). However, such effect was not observed in the UEC, as the ability to visualise extra canals (e.g. the MB2) was shown to be severely compromised, regardless of the additional diagnostic aids used (31).

#### Chemomechanical debridement

Recent studies explored the effect of MIEC on instrumentation and disinfection of the root canal system by measuring the proportion of untouched canal area and the amount of post-debridement bacterial load (6-8, 20, 26, 32, 42, 43, 45, 47, 55). A few studies reported that the MIEC compromised instrumentation in the mandibular molars, leaving a higher proportion of untouched canal area than TEC (20, 55). In addition, a higher percentage of the pulp tissue remnants in the pulp chamber was also found in the mandibular molars, which could potentially affect the thorough disinfection of the root canal system (26). Some solutions have been suggested to overcome the shortcomings by incorporating activated irrigation protocols or extending the outline of the access cavity to accommodate the oval-shaped canal (20, 26).

In contrast, other studies reported no significant differences in the percentage of untouched canal area in the maxillary and mandibular molars (6, 7, 42, 47), maxillary premolars (8) and mandibular incisors (32, 43). Although most recent studies by Barbosa et al. and Tüfenkçi et al. demonstrated similar efficacy of bacterial elimination between the CEC and TEC in the mandibular molars (45, 55), Vieira et al. showed a significantly higher number of samples with bacteria-positive cultures in the CEC group when compared to the TEC group (32). In the latter study, similar proportions of unprepared areas were also found between the two groups (32). In summary, results comparing the efficacy of disinfection between the TEC and CEC remain controversial, while no advantage in canal instrumentation was rendered by the CEC design.

#### Procedural accident

MIEC is generally technically challenging and demands advanced skills and experience (46). The presence of coronal dentinal interference is an obstacle that may hinder the instrument's ability to conform to the original canal anatomy. This, in turn, increases the chance of iatrogenic errors, including canal transportation consequential to unwanted straightening of the canal curvature, canal perforation and apical extrusion (30). The increased deviation from the original canal pathway might be attributed to the reduction in the centring ability of the instrument around the curvature, hence the need for more pecking motion during instrumentation in the presence of coronal interference (30). Overall, discordant results have been reported, with some studies demonstrating a higher incidence of apical extrusion or canal transportation (7, 30, 39, 52), while others did not (6, 42, 43, 47, 55).

#### Instrument fracture

Only one study has investigated the influence of the UEC on the cyclic resistance of two types of NiTi instruments, namely RECIPROC R25 (R25, VDW, Munich, Germany) and RECIPROC Blue R25 (R25, VDW, Munich, Germany), using the TEC for comparison (53). It was reported that both files exhibited a lower cyclic fatigue resistance in the lower molars when the teeth were accessed with the UEC design when compared to TEC. The explanation was that UEC access increases the angle of canal curvature, resulting in greater stress along the file at the points of curvature, compared to TEC. Since the study only tested two file systems produced by the same manufacturer, the performance of other file systems on teeth prepared with UEC has yet to be explored. On the other hand, studies that investigated the effects of MIEC on the fracture resistance of ETT did not observe any increased incidence of instrument fracture (20, 30, 39, 42, 43). In these studies, merely one single system of NiTi instrument was used by the experienced endodontists with the aid of OM (20, 30, 39, 42, 43). Nevertheless, the use of flexible NiTi instruments in MIEC seems advantageous in lowering the incidence of instrument fracture.

#### Root canal filling quality

Root canal obturation using the single cone and warm vertical compaction technique in teeth prepared with CEC might result in an increased number of voids, as shown by a study performed on the mandibular incisors (7). The single cone technique on the mandibular premolars also faced the same obstacle, as reported in another study (60). This might be due to the constricted access cavity hindering the matching single cone's proper placement, which prompted the authors to recommend using the warm lateral compaction technique instead (60). However, other studies found no significant differences in the quality of the root canal filling in terms of the number of voids when the single cone technique was used (8, 24). The inconsistencies in findings may be explained by the difference in the canal morphology investigated in the individual studies. For instance, the mandibular incisors often have oval-shaped canal morphology that possibly added to the complexity of root canal shaping, disinfection, and filling procedures (78).

#### Quality of the post-endodontic restoration

The role of quality of the coronal restoration towards successful root canal treatment outcome has been emphasised in the literature (79). One study evaluated the effect of the UEC on gaps and voids formation in the composite restorations placed on endodontically treated maxillary premolars and showed that the teeth prepared with UEC presented an increased number of voids in bulk fill composite, albeit no increase in gap formation was detected (54). It might be related to the challenges faced in handling the filling materials in a constricted access cavity.

#### Aesthetics

In the anterior teeth, MIEC is usually created from the incisal edge to partial deroofing of the pulp chamber, leaving the pulp horns intact. As a result, the ability to completely remove pulp remnants from the pulp chamber and adequate placement of the intracoronal bleaching agent into the constricted access cavity is hindered (75). Additionally, excess root canal filling or remnant in the access cavity have been attributed to tooth discolouration (75), and such unaesthetic side effect appeared to be more evident when the CEC design was adopted (54).

Marchesan et al. (40) addressed the impact of CEC on non-vital bleaching of the discoloured anterior teeth. The authors stated that when 35% carbamide peroxide was used as the bleaching agent, the discoloured maxillary central incisors in the CEC group could not regain the pre-staining lightness value, while the TEC group did not show the same phenomenon (40). Hence, the clinical application of MIEC in the anterior aesthetic zone is not without problem, in addition to the fact that no improvement in the fracture resistance was evident in the ETT of the maxillary and mandibular incisors (20, 43).

#### Treatment time

Several authors have reported significantly longer canal preparation time for teeth accessed with the CEC or UEC (6, 8, 39, 40, 52). For example, Marchesan et al. (39) measured the treatment time used in the CEC and TEC and found that a 2.5-fold greater time was needed for canal instrumentation in the former design. Hence, an increase in the treatment duration may be regarded as an additional disadvantage of MIEC.

#### Clinical application

Until now, most studies have been performed on intact teeth *ex vivo*, limiting their clinical application to coronally intact teeth requiring root canal treatment due to pulpal or periapical pathology, such as those secondary to orthodontic movement, luxation injuries without crown fractures and dens evaginatus, etc. Meanwhile, most teeth requiring root canal treatment are either carious or previously restored, making the clinical application of the MIEC on such teeth worthy of further research.

The ability of MIEC to identify cracks might also be hindered owing to the reduced illumination in smaller access cavities and areas of undercuts, such as those beneath the preserved pulp horns. In addition, vital or necrotic pulp tissue remnants and debris may also obscure the presence of cracks and indiscriminately takes up methylene blue staining used for crack detection.

The authors anticipate that procedural challenges, such as canal location, instrumentation and disinfection, are likely to be greater for the teeth studied in the *in vivo* than *ex vivo* studies. Besides, most of the previous studies were conducted on the CEC. Thus, this review focuses largely on the CEC, necessitating due caution before directly comparing the performance of various MIEC designs.

The authors suggested that a few clinical approaches may be adopted in MIEC to overcome the problems in chemomechanical debridement of the root canal system, which include:

1. Increase the concentration of the disinfectant used as irrigant;
2. The use of irrigant agitation techniques;
3. Increase the time spent on chemical disinfection;
4. The use of heat-treated NiTi files with improved flexibility and fatigue resistance;
5. The placement of calcium hydroxide as intracanal medicament;
6. The use of the retrograde surgical tip to access and debride the areas beneath the pulp horns; and,
7. The use of an operating microscope.

## CONCLUSION

Although the importance of preserving tooth structure appears self-evident, it can be concluded from this literature review that the complete transition to MIEC has yet to be validated. Therefore, the application of MIEC in clinical practice requires critical consideration by weighing the risks and benefits of the TEC and MIEC. Furthermore, the currently available evidence is insufficient to support the use of MIEC indiscriminately in routine endodontic practice.
